# Robust Hydrogel Adhesive with Dual Hydrogen Bond Networks

**DOI:** 10.3390/molecules26092688

**Published:** 2021-05-04

**Authors:** Zhiqiang Jiang, Ya Li, Yirui Shen, Jian Yang, Zongyong Zhang, Yujing You, Zhongda Lv, Lihui Yao

**Affiliations:** School of Materials Science and Chemical Engineering, Ningbo University of Technology, 201 Fenghua Road, Jiangbei, Ningbo 315211, China; liya@nbut.edu.cn (Y.L.); elfreda_shen@163.com (Y.S.); yangj@iccas.ac.cn (J.Y.); zzy_whut@126.com (Z.Z.); lvzd_nbut@163.com (Z.L.); ylhttzj@hotmail.com (L.Y.)

**Keywords:** hydrogels, tissue adhesive, hydrogen bonds, stretchable, notch-insensitive

## Abstract

Hydrogel adhesives are attractive for applications in intelligent soft materials and tissue engineering, but conventional hydrogels usually have poor adhesion. In this study, we designed a strategy to synthesize a novel adhesive with a thin hydrogel adhesive layer integrated on a tough substrate hydrogel. The adhesive layer with positive charges of ammonium groups on the polymer backbones strongly bonds to a wide range of nonporous materials’ surfaces. The substrate layer with a dual hydrogen bond system consists of (i) weak hydrogen bonds between *N*,*N*-dimethyl acrylamide (DMAA) and acrylic acid (AAc) units and (ii) strong multiple hydrogen bonds between 2-ureido-4[1H]-pyrimidinone (UPy) units. The dual hydrogen-bond network endowed the hydrogel adhesives with unique mechanical properties, e.g., toughness, highly stretchability, and insensitivity to notches. The hydrogel adhesion to four types of materials like glass, 316L stainless steel, aluminum, Al_2_O_3_ ceramic, and two biological tissues including pig skin and pig kidney was investigated. The hydrogel bonds strongly to dry solid surfaces and wet tissue, which is promising for biomedical applications.

## 1. Introduction

Hydrogel adhesives that forms quick, strong, and reversible adhesion to biological tissues are attractive for actuators [1,2], electronics [3], and biomedical applications [4,5,6]. They are elastic and flexible and provide a biocompatible interface [7]. However, because hydrogel contains a large volume of water, and because their adhesion to wet solid surfaces takes place by various subtle factors like local physical and chemical interactions including ion interactions [8], hydrogen bonding [9,10], metal coordination [11], topology [12,13,14], and matrix mechanical properties, to achieve strong wet adhesion remains challenging. For example, fibrin glue [15] and polyethylene glycol-based adhesives [16] form covalent bonds with tissues, but the poor mechanical strength of the hydrogels restrains practical use [17].

Marine mussels bond fast and strongly to rock surfaces in the seawater. They illustrate the key factors for strong wet bonding. The catechol structures from the precursor 3,4-dihydroxyphenyl-l-alanine (DOPA) in mussel foot proteins have been identified to mediate the robust bonding [18]. Catechol forms coordination bonds with oxides on the surface of mineral substrates [19]. These strong interactions give rise to a high interfacial attraction. Catechols form H-bonds [20,21] and can be oxidized into quinones to crosslink the proteins. They also coordinate with metal ions like Ca^2+^, Zn^2+^, and Fe^3+^ [22,23], which enhances cohesive strength. Both the interface and matrix play a critical role in achieving tough bonding. These results show that hydrogel adhesives should not only have strong microscopic interfacial interaction with the substrate but also tough matrix macroscopic mechanical properties to sustain the load and dissipate the energy.

Inspired by the mussels’ biological events, several approaches have been followed to improve the hydrogel adhesion by optimizing the surface chemistry and hydrogel matrix. Biomimetic moieties containing catechol units [24,25,26], polydopamine [27,28], polyelectrolytes [8,29,30], and reactive chemical groups like *N*-hydroxysuccinimide (NHS) [31,32] were incorporated into the hydrogels to enhance adherence. Barrett et al. reported that the hydrogel of PEO-PPO-PEO endcapped with catechols [33,34] adhered to hydrated tissue surfaces. Lapitsky and co-workers prepared a gel from cationic polyallylamine crosslinked by phosphate anions [35,36]. Recently, hydrogels with high strength and toughness, e.g., double network (DN) hydrogels, were reported as the substrate for the hydrogel adhesive. Yuk et al. [37] chemically anchored the polymer chains of an alginate-polyacrylamide (Alg-PAAm) DN hydrogel to solid surfaces using silane modification and EDC chemistry. Li et al. [38] reported a hydrogel adhesive by grafting polymers onto the surface of an Alg-PAAm DN hydrogel. These tough Alg-PAAm DN hydrogels worked as structural support and contributed to the energy dissipating in the peeling process, which led to high adhesion.

In this study, we designed a double-layer (DL) hydrogel with a thin adhesive layer on a tough hydrogel substrate. A surface layer with positive charged ammonium groups (2-(acryloyloxyethyl) trimethylammonium chloride, AEtMA-Cl) was prepared. In contrast to prior approaches that used DN hydrogels, we synthesized a tough hydrogel that consisted of dual hydrogen bond networks. One network involved acrylic acid (AA) and *N*,*N*-dimethyl acrylamide (DMAA) and the other one involved UPy moieties that form quadruple hydrogel bonds. Such a type of dual hydrogen-bond networks rendered the matrix substrate a high toughness [39]. The hydrogels’ adhesive properties to different material surfaces and biological tissues were studied.

## 2. Results and Discussion

### 2.1. Synthesis of the Double-Layer Hydrogel

**Figure 1:**
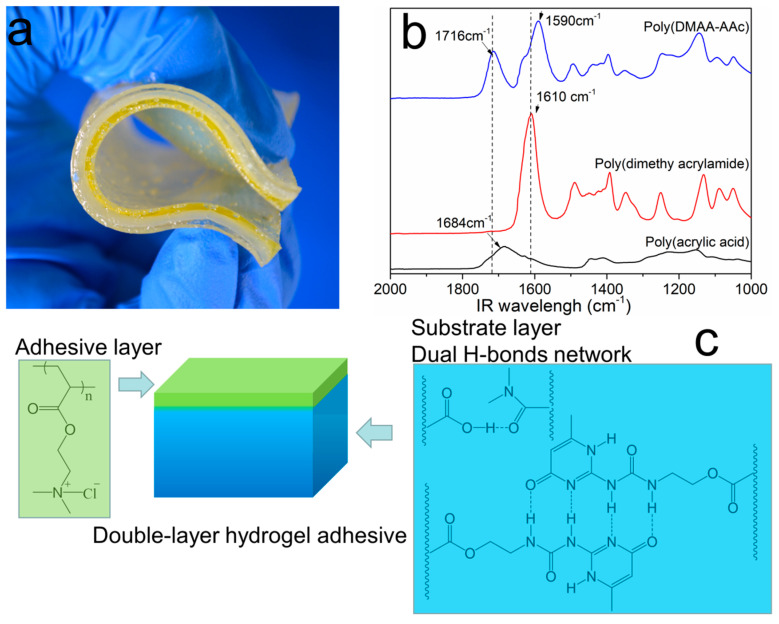
(**a**) Actual image of a DL hydrogel sample. The top adhesive layer was yellow-stained. (**b**) IR spectra of polyacrylic acid, poly(*N*,*N*-dimethyl acrylamide), and poly(DMAA-co-AA). (**c**) Schematic representation of dual hydrogen-bond networks.

The adhesive consisted of two layers, the bottom layer and the top adhesive layer. The hydrogel substrate was synthesized by free radical copolymerization of *N*,*N*-dimethyl acrylamide, acrylic acid, and 2-(3-(6-methyl-4-oxo-1,4-dihydropyrimidin-2-yl) ureido) ethyl acrylate in aqueous solution. Under UV irradiation, the solution changed into a gel within 5 min. At that time the bulk became a gel, but its surface remained viscous with monomers not fully consumed. The monomer solution (2-(acryloyloxyethyl) trimethylammonium chloride) to build the adhesive layer was added quickly onto the top of the substrate layer, and the polymerization was continued under UV irradiation. These monomer molecules bearing ammonium groups were used to build the adhesive layer because such positively charged moieties favorably interact with the carboxylate groups of acrylic acid entities, strengthening interfacial bonding. The reactant-carrying ammonium groups diffused into the soft and viscous liquid phase of the superficial layer and then polymerized. After the reaction was continued for another 60 min, the top layer hydrogel was formed and merged with the substrate layer to give one single gel (Figure 1a).

### 2.2. Hydrogen Bonds between DMAA and AAc Units

The dimethyl amide group is a strong hydrogen-bond acceptor, whereas acrylic acid is a potent hydrogen-bond donor. The amide groups on PDMAA are able to form hydrogen bonds with the carboxylic acid groups (Figure 1b). To confirm the hydrogen bonds between AA and DMAA, we prepared the poly(acrylic acid), poly(*N*,*N*-dimethyl acrylamide), and their copolymers. In the FT-IR spectra, the peaks observed were at 3350 cm^−1^ corresponding to the -OH stretching of the carboxylic acid unit. Bending vibrations of CH_2_ groups into the main polymer chains give an absorption band at 1460 cm^−1^. The absorption band located at 2935 cm^−1^ was ascribed to stretching vibrations of the CH groups of the acrylate units. The absorbance at 1610 cm^−1^ was attributed to the C=O groups of the acrylamide units. The formation of hydrogen bonding and other molecular interactions affect the position of the involved peaks. The wavenumber of the C=O peak decreased from 1610 to 1590 cm^−1^. The carbonyl stretching of carboxyls of poly(acrylic acid) at 1684 cm^−1^ was shifted to longer wavenumbers (1716 cm^−1^) as in the poly(DMAA-AAc). These results indicated an interaction between the carboxylic groups of AAC and the amide groups of DMAA. The binding energy between hydrogen bonds from a series of donors and acceptors was reported by the quantum chemistry DFT method [40]. The hydrogen-bonding energy between the amide acceptor and the hydroxyl donors was −4.51 kcal/mol. The hydrogen-bonding constant (K_dim_) was calculated according to the following equation:

log K = −0.892 × ∆*E*_ΗΒ_ − 2.630 (1)

so,

log K = 1.66, K = 39.8 M^−1^ (2)

### 2.3. Hydrogen Bonds between UPy Units

UPyEA moieties form quadruple hydrogen-bonded DDAA dimers, both in the solid state and in solutions as confirmed by ^1^H and ^13^C NMR spectroscopy [41,42,43]. The hydrogen-bonding energy (∆G°dim) and equilibrium constant of UPy were calculated to be −44 kJ/mol and 6 × 10^7^ M^−1^, respectively, in CDCl_3_ [43]. So, the quadruple hydrogen bonds from the UPyEA moieties and the hydrogen bonds from the AAC and DMAA formed two hierarchical hydrogen-bond networks on different strength levels (Figure 1c). The quadruple-hydrogen bonds between UPy units were strong and the hydrogen bonds between DMAA and AAc were relatively weak and more dynamical. They dissociated and re-associated continuously under stress, acting as sacrificial bonds to dissipate the excess energy.

### 2.4. Mechanical Properties

**Figure 2:**
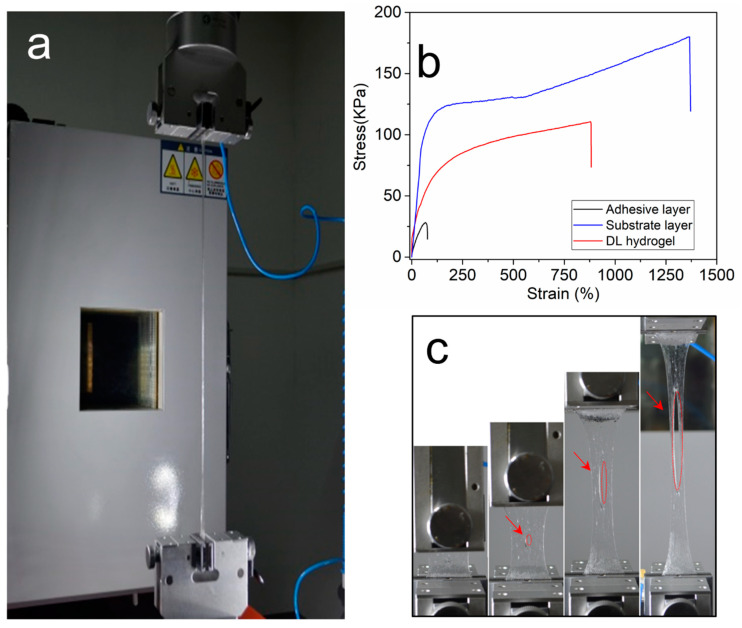
Mechanical properties of the hydrogels. (**a**) Photographs of a hydrogel under tensile test (v = 25 mm/min). (**b**) Tensile stress–strain curves of the AEtMA-Cl layer, substrate layer, and DL hydrogel as prepared. (**c**) Photographs of the hydrogel during a notch tensile test (v = 25 mm/min).

During the mechanical testing, the hydrogels’ properties in the tensile mode were studied (Figure 2a). Figure 2b shows stress–strain curves for the top poly(AEtMA) layer, the substrate layer, and the DL hydrogel samples. The poly(AEtMA) layer alone was mechanically weak. It had a maximum stress of 27.8 kPa corresponding to a maximum strain of 68%. The substrate layer was tough and stretchable with a maximum stress of (180 ± 23) kPa at the maximum strain of (1370 ± 74)%. For comparison, the stress at failure of the DN hydrogel (consisting of a mixture of ionically crosslinked alginate and covalently crosslinked polyacrylamide and used as structural support for hydrogel adhesive) was 156 kPa [44]. The two networks of the weak poly(AA-co-DMAA) structure and the tough Upy-based matrix, both physically crosslinked via H-bonding, contribute synergistically to the robust mechanical strength of the final hydrogel. Thus, the tensile strength of (110 ± 11) kPa and the maximum strain of (880 ± 65)% measured for the DL hydrogel were 3.9 and 12.9 times higher, respectively, than those of the primary AEtMA-Cl-based layer.

Air voids or cracks in the hydrogel generally downgrade the mechanical properties. If a small crack exists, it will quickly propagate and multiply in the hydrogel under stress, which leads to macroscopic failure. By introducing dual hydrogen bonds into the polymer network, our hydrogel displayed an excellent notch-insensitive property (Figure 2c). A crack of 6 mm in length was made in the DL hydrogel specimen (L = 25 mm, W = 18 mm), then the gel was stretched to a strain of 840%. The notch remained extremely stable as the hydrogel toughness was enhanced by the dual hydrogen-bond networks across the front of the crack.

### 2.5. Adhesion Properties

**Figure 3:**
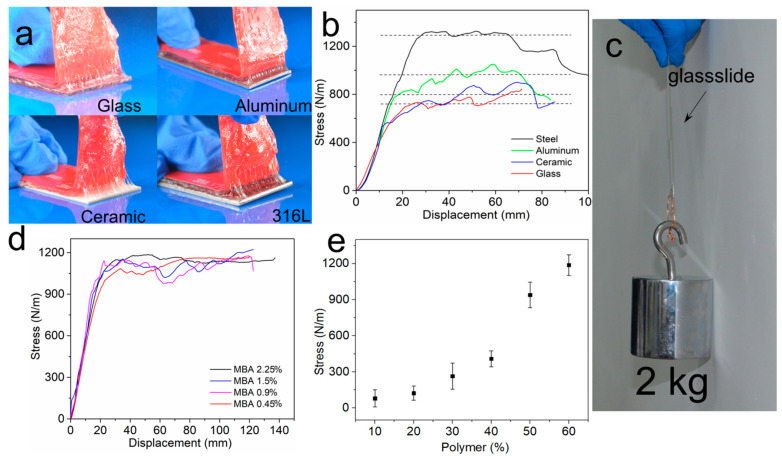
(**a**) Photos of the peeling process of the AEtMA-Cl-based layer physically attached on four solid substrates: glass, aluminum, Al_2_O_3_ ceramic, and 316L stainless steel. (**b**) Stress–displacement curves of the layer on the different surfaces. (**c**) Photograph of AEtMA-Cl-based hydrogel between two glass slides bearing a 2 kg load. (**d**,**e**) The adhesion of AEtMA-Cl hydrogels with different crosslinker contents and polymer concentrations by 90° peeling tests.

We investigated the adhesion of the AEtMA-Cl-based layer to four different types of materials: glass, 316L stainless steel, aluminum, and Al_2_O_3_ ceramic. The samples were prepared by hydrogel formation directly on the surfaces of materials. A commercial 3M tape was applied onto the hydrogel surface by force to prevent the hydrogel from stretching significantly during tests (Figure 3a). When the hydrogel was peeled off from the surface, the tough substrate layer was deformed following the adhesive layer and thus contributing to the energy dissipation [45,46]. The adhesive strength of hydrogel was as high as 1290 ± 84 N/m and 960 ± 75 N/m on the 316L stainless steel and the aluminum surfaces, respectively (Figure 3b). The adhesive strength on the Al_2_O_3_ ceramic (805 ± 64 N/m) was slightly higher than on the glass (723 ± 77 N/m) because Al_2_O_3_ slides exhibit more pronounced surface roughness. The hydrogel showed very high adhesive strength, and these results were superior to Scotch tape (ca. 180 N/m) and duct tape (ca. 400 N/m) [47]. As shown in Figure 3c, a hydrogel-glass interface was prepared by directly polymerizing the reactant AEtMA-Cl solution between two glass surfaces via UV irradiation. The shear adhesive strength of the AEtMA-Cl gel between the glasses was so strong as to lift 2.0 Kg (≈4000 times the gel’s weight).

We also studied the effect of a crosslinker (*N*,*N*’-methylene bisacrylamide) in the AEtMA-Cl-based layer with the following weight percentages with respect to the AEtMA-Cl monomer: 0.45, 0.90, 1.50, and 2.25%. However, the results did not show noticeable changes (Figure 3d). AEtMA-Cl units are hydrophilic and highly swollen in water. The increase of crosslinker content in this range probably did not change the surface properties. On the other hand, we found that water content played a critical role in determining the adhesive strength of the AEtMA-Cl-based hydrogel (Figure 3e). Thus, water can be a good plasticizer and can enhance the adhesive strength ((1210 ± 74) N/m) when its concentration reaches 40%. When water content increased to 50%, the cohesive strength slightly decreased (987 ± 91 N/m). By continuing this ascendant tendency to attain 90% water content, the adhesive strength diminished substantially (87 ± 11 N/m).

### 2.6. Attachment of Adhesive to Tissues

**Figure 4:**
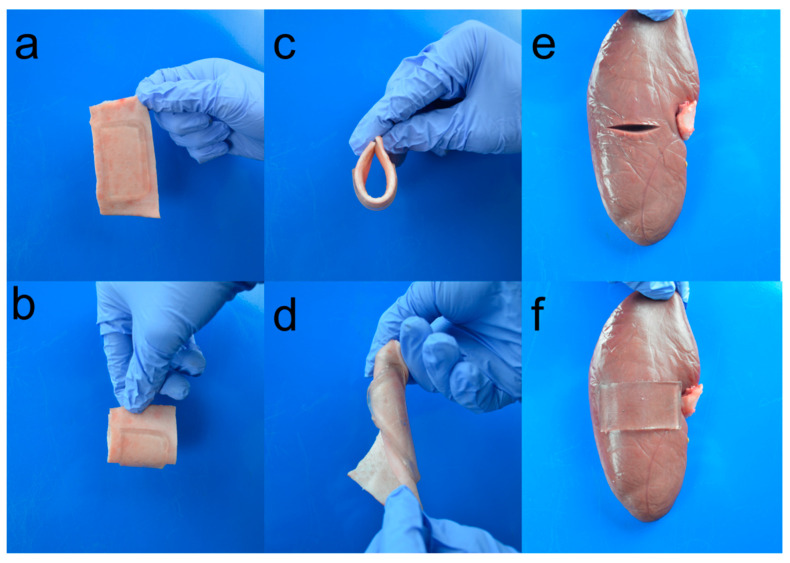
Photographs of the hydrogel bonded to pig skin (**a**) in the state of dangling, (**b**,**c**) being bent, and (**d**) twisted. Photographs of hydrogel adhesive adhered to pig kidney. (**e**) A notch was made in the pig kidney. (**f**) Hydrogel bonds to the pig kidney surface and connects the two sides.

To evaluate the potential application of the hydrogel for closure of a skin wound, we investigated its adherence to pig skin. A rectangle pig skin was cut and cleaned by ethanol solution (70% v/v in distilled water) to remove the grease on the surface. Then, the hydrogel was quickly applied to the surface. The gel adhered to the skin tightly when the skin was bent and twisted (Figure 4a–d). Despite the huge strain caused by the substrate pig skin, the gels remained intact. The hydrogel was also used to bond a pig kidney. One deep notch was made on the surface of the fresh pig kidney horizontally (Figure 4e). The notch edges were held together and the hydrogel was applied under slight pressure for 30 s to cover the notch mechanically closed. As a result, the gel stuck to the kidney immediately and the notch was closed (Figure 4f). The hydrogel bonded the tissue and the adhesive strength was so strong that when it was peeled off, the fibrous capsule on the kidney surface was peeled with the hydrogel. These results suggest that the hydrogel achieved robust hydrogel–tissue bonding.

The interfacial adhesion of this double-layer hydrogel depends on the polymer concentration in the AEtMA-Cl-based hydrogel. The adhesion strength increased by 14.8 times when the polymer concentration increased from 10 to 60%. When it was 60%, the hydrogel’s adhesion strength was comparable to that of the chemically modified Alg-PAAm DN hydrogel (ca. 1100 N/m) [37]. Alg-PAAm DN hydrogel is a typical tough hydrogel with two chemically crosslinked networks. One network is strong and rigid and the other is soft. The soft network breaks under load and dissipates energy. In this double-layer hydrogel adhesive, the substrate hydrogel had two distinct hydrogen-bond networks. The weak one dissociates under load and endows the substrate gel with toughness. The tough substrate and highly adhesive top layer worked synergistically to gain robust bonding.

Although the hydrogel is not biodegradable, it is a promising hemostat material. It is flexible, stretchable, and notch-insensitive, which is beneficial to skin wound treatment. Fast bonding and instant bleeding control can be obtained by quickly applying this gel to the wound lesion. Enzymes like thrombin [48] and antibacterial nanoparticles like ZnO [49] can be incorporated into the gel to promote wound healing.

## 3. Experimental

### 3.1. Materials

AEtMA-Cl, *N*,*N*-dimethylacrylamide (98%), acrylic acid, *N*,*N*’-methylenebis(acrylamide) (MBA), α-ketoglutaric acid, and Triton X-100 were purchased from Sigma-Aldrich (Tansoole, China) and used without further purification. 2-(3-(6-methyl-4-oxo-1,4-dihydropyrimidin-2-yl) ureido) ethyl acrylate was synthesized according to a protocol reported elsewhere [48].

### 3.2. Synthesis of DL Hydrogel

The P(AA-AAm) DL adhesive samples were fabricated as follows. First, the hydrogel substrate was synthesized by free radical copolymerization of *N*,*N*-dimethylacrylamide (9.26 g), acrylic acid (6.74 g), 2-(3-(6-methyl-4-oxo-1,4-dihydropyrimidin-2-yl) ureido) ethyl acrylate (0.62 g), surfactant Triton X-100 (0.1 g), and α-ketoglutaric acid (0.1 g) as the photoinitiator in distilled water (23.2 mL). The mixture was sonicated for 30 min to give a clear solution. The aqueous solution (40 mL) was purged with nitrogen gas to replace O_2_ in the system for 30 min. Then, the solution was cast into a cubic plastic box (11 × 9 × 1 cm^3^), and the polymerization reaction was initiated by UV irradiation (λ = 365 nm). The reactant solution became a viscous solid within 2–3 min, then a 10 mL solution consisting of AEtMA-Cl (6.0 g), *N*,*N*’-methylene bisacrylamide (MBA) (0.05 g), and distilled water (4 mL) was added onto the substrate surface and was irradiated by UV for another 1 h. After the double-layer hydrogel was formed, the samples were sealed in a plastic bag and then placed into an oven overnight at 50 °C.

### 3.3. FT-IR

The samples were synthesized in aqueous solutions (1 wt%), coated on KBr, and dried in a vacuum oven to the constant weight. IR spectra were acquired in the wavenumber range of 1200 to 4000 cm^−1^ (FT-IR instrument, Nicolet 6700, Madison, WI, USA).

### 3.4. Mechanical Properties

Rectangular specimens (30 × 15 × 1.5 mm^3^) coated with silicone oil were stretched by an Instron machine (model 3342 with a load cell of maximum 2000 N). For notched samples, an edge crack of 6 mm length was cut in the middle of the specimen by using a razor blade. The stretch rate was fixed at 25 mm·min^−1^.

### 3.5. Interfacial Toughness Measurement

The peeling strength between the hydrogel and substrates was measured using a standard 90° peeling test (ASTM D6862) with a mechanical testing machine (Instron, MA, US, model 3342 with a load cell maximum of 2000 N). A sample with a gel layer coating thickness of 1.0 mm was cut to specific dimensions (length: 75 mm and width: 25 mm). To prevent stretching of the adhesive gel, 3M VHB4910 tape with the release film was bonded onto the surface of the AEtMA-Cl-based hydrogel. The peeling tests were performed at a constant peeling velocity of 25 mm·min^−1^. When the measured force reached a plateau, the value was treated as the peeling force. The peeling strength was determined by dividing the peeling force by the width of the sample.

### 3.6. Attachment of DL Adhesive to Tissues

For the use of pig heart and kidney wound dressing, a notch (20 mm) was made with a blade. The DL hydrogel adhesive of 25 mm width was applied with compression for 30 s.

## 4. Conclusions

In summary, we successfully designed a double-layer hydrogel adhesive using a biomimetic priming adhesive layer to enhance bonding performance and a substrate layer to enhance the matrix strength and toughness. Our simple method relied on the partial diffusion of the superficial adhesive layer into the tough substrate layer and merging them into one single bulk hydrogel. The hydrogel substrate was demonstrated to be stretchable, tough, and notch-insensitive. The AEtMA-Cl-based layer bonded strongly to the various nonporous surfaces, whereas the substrate provided strong structural support. Tough bonding to various solid surfaces like glass, Al_2_O_3_ ceramic, and stainless steel was successful. The hydrogel also bonded to tissues like pig skin and pig kidney. This hydrogel has a useful potential for designing biomedical materials.

## Data Availability

The data presented in this study are available on request from the corresponding author.

## Sample Availability

Samples of DL hydrogels are available from the authors.
